# Detailed investigation of the lesion formation with a novel contact force sensing catheter with a mesh‐shaped irrigation tip

**DOI:** 10.1002/joa3.12835

**Published:** 2023-02-27

**Authors:** Kazuhisa Matsumoto, Daisuke Kawano, Wataru Sasaki, Naomichi Tanaka, Masataka Narita, Hitoshi Mori, Kenta Tsutsui, Yoshifumi Ikeda, Takahide Arai, Shintaro Nakano, Ritsushi Kato, Kazuo Matsumoto

**Affiliations:** ^1^ Department of Cardiology Saitama Medical University International Medical Center Hidaka Japan

**Keywords:** contact force, ex vivo experimental model, lesion size, radiofrequency ablation, TactiFlex catheter

## Abstract

**Background:**

Recently, a novel contact force (CF) sensing catheter with mesh‐shaped irrigation tip (TactiFlex SE, Abbott) was invented and is expected to be useful for safe and effective radiofrequency ablation. However, this catheter's detailed characteristics of the lesion formation are unknown.

**Methods:**

With an in vitro model, TactiFlex SE and its predecessor, FlexAbility SE, were used. A cross‐sectional analysis of 60 s lesions (combination of various energy power settings [30, 40, and 50 W], and CFs [10, 30, and 50 g]) and longitudinal analysis (combination of various powers [40 or 50 W], CFs [10, 30, and 50 g] and ablation times [10, 20, 30, 40, 50, and 60 s]) of both catheters were analyzed and compared.

**Results:**

One hundred eighty RF lesions were created in protocol 1 and 300 in protocol 2. The lesion formation, impedance changes, and steam pops characteristics were similar between the two catheters. Higher CFs were related to higher incidences of steam pops. A nonlinear, time‐dependent increase in the lesion depth and diameter was observed for all power and CF settings, and linear, positive correlations between the RF delivery time and lesion volume were observed for all power settings. Compared with 40 W, a 50 W ablation created greater lesions. Longer durations with higher CF settings had a higher steam pop incidence.

**Conclusions:**

The lesion formation and incidence of steam pops with TactiFlex SE and FlexAbility SE were similar. A 40 or 50 W ablation with careful CF control not to exceed 30 g in addition to monitoring impedance drops was required to safely create transmural lesions.

## Graphical abstract

Lesion formation using a TactiFlex catheter.

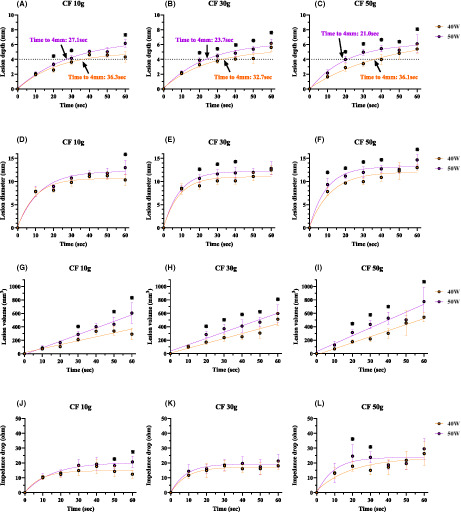

## 1 INTRODUCTION

Radiofrequency (RF) ablation has become a well‐established treatment of cardiac arrhythmias.^1^, ^2^ Modern ablation catheters are equipped with an irrigation technology, which cools the ablation electrode to prevent overheating and therefore the formation of char and thrombi.^3^, ^4^ The previous irrigation technology, for example, the 6‐hole version (that of TactiCath™ SE, Abbott), required a high flow rate at 30 mL/min and was even sometimes insufficient to effectively cool the ablation tip.^5^ Recently, the latest iteration with a mesh‐shaped tip design has been introduced (FlexAbility™ SE, Abbott).^6^, ^7^ Thanks to the novel tip design, the irrigation flow rate of these products has been substantially reduced from 30 to 13 mL/min while it promises a reduction in the incidence of char and thrombus formation,^5^ and a recent study reported that mesh‐shaped irrigation catheters are highly effective in preventing procedure‐related ischemic complications.^8^ However, the FlexAbility™ SE catheter does not have a contact force (CF) technology, which helps predict the lesion size and prevent steam pops caused by a high catheter contact with the local tissue.^9^, ^10^, ^11^ A previous study reported that the FlexAbility™ SE catheter had a high recurrence rate of atrial tachycardia after atrial fibrillation (AF) ablation and required a longer ablation time than the TactiCath™ SE catheter, because of the absence of a CF sensor.^12^ In more recent days, a novel CF sensing catheter with a mesh‐shaped irrigation catheter (TactiFlex™ SE, Abbott) has been invented and is available for clinical practice. Although this novel catheter helps us to determine the CF information with effective cooling of the distal tip, the detailed influence of the CF sensing equipment with a mesh‐shaped tip on the lesion formation with these catheters remains unknown. In this study, we systematically assessed the lesion formation characteristics of the two mesh‐shaped irrigation catheters (TactiFlex™ SE and FlexAbility™ SE) in an ex vivo experimental model.

## 2 METHODS

### 2.1 Ex vivo experimental model

**FIGURE 1:**
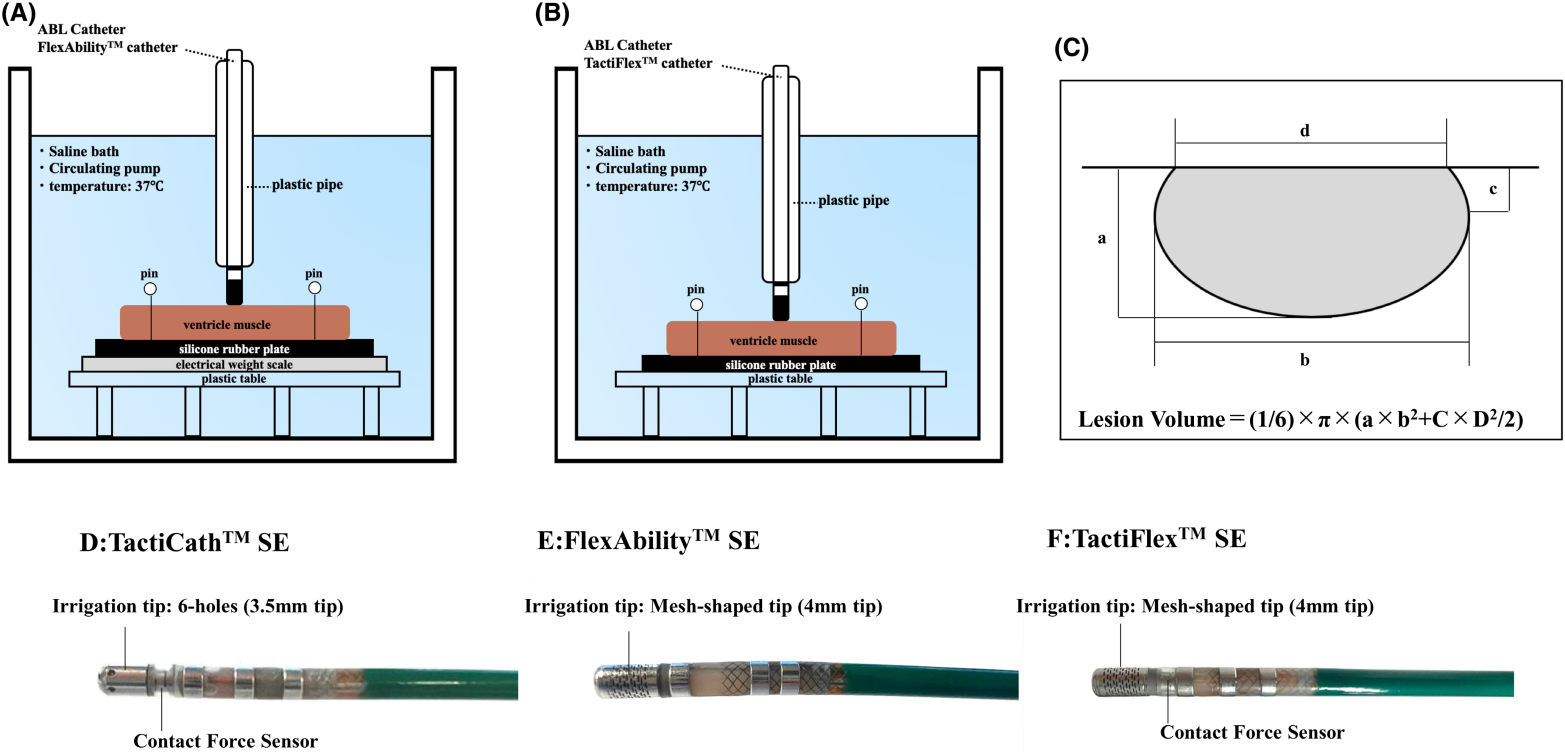
(A and B) An in vitro experimental model. A swine left ventricle slice was fixed on a plate in a bath filled with saline, which was circulated and heated by a peristaltic pump with a thermostat. An ablation catheter was stabilized manually in a plastic pipe oriented perpendicular to the tissue. (A) An electrical weight scale was placed below the rubber plate to monitor the tissue contact force because the FlexAbility™ SE catheter has no contact force sensing. (C) The maximum depth (a), maximum diameter (b), depth at the maximum diameter (c), and surface maximum diameter (d) of the lesion were measured. The lesion volume was calculated as the volume = (1/6) × π × (a × b^2^ + c × d^2^/2). (D) A catheter image of the TactiCath™ SE catheter, which has a 3.5 mm tip with 6 irrigation holes and a contact force sensor. (E) A catheter image of the FlexAbility™ SE catheter, which has a 4.0 mm tip with a mesh‐shaped irrigation tip without a contact force sensor. (F) A catheter image of the TactiFlex™ SE catheter, which has a 4.0 mm tip with a mesh‐shaped irrigation tip and contact force sensor.

The experimental protocol was performed as previously described (Figure 1A,B).^13^, ^14^ In short, a fresh swine left ventricle slice was fixed on a plate in a bath filled with saline, which was circulated and heated by a peristatic pump with a thermostat. The pool temperature was kept at 37°C. Either the FlexAbility™ SE or TactiFlex™ SE ablation catheter was stabilized manually through a plastic pipe and placed perpendicularly to the tissue. To monitor the CF with the FlexAbility™ SE catheter, which cannot monitor the CF information by itself, an electrical weight scale (Kitchen scale KD‐187; Tanita Co.) was placed below the rubber plate.^15^ The irrigation flow rate was set at 13 mL/min for both catheters.

### 2.2 RF lesion assessment

The lesion border was defined as a change in the tissue color. The maximum depth (a), maximum diameter (b), depth at the maximum diameter (c), and surface maximum diameter (d) of the lesion were measured. Then, the lesion volume and lesion surface area were calculated as the volume = (1/6) × π × (a × b^2^ + c × d^2^/2) (Figure 1B), surface area = π × d/2 × e/2).^13^

### 2.3 Study protocol

#### 2.3.1 Protocol 1: Comparison of the lesion formation between a contact force sensing catheter and noncontact mesh‐shaped irrigation catheter under a fixed time setting

Protocol 1 was designed to compare the cross‐sectional analyses of the ablation characteristics over a fixed time between TactiFlex™ SE and FlexAbility™ SE. The experiment was performed with a combination of various energy powers (30, 40, 50 W) and CF steps (10, 30, and 50 g). RF energy was fixed at 60 s. The lesion formation, ablation parameters, and incidence of steam pops with TactiFlex™ SE and FlexAbility™ SE were analyzed and compared.

Steam pops were defined as audible pops. An impedance drop was defined as the difference between the initial impedance and lowest impedance during an RF application. Even when a steam pop occurred, the RF energy delivery was continued for 60 s. The experiments at each setting were repeated 10 times, and all the data were analyzed.

#### 2.3.2 Protocol 2: Detailed investigation of the lesion formation with a novel contact force sensing catheter with a mesh‐shaped irrigation tip under a fixed power setting

Protocol 2 was designed to investigate the longitudinal evolution of the lesion size with the TactiFlex™ SE catheter. A previous study reported that the lesion size with FlexAbility™ SE was smaller than that with TactiCath™ SE, arguably because of the difference in the tip length (the tip lengths of FlexAbility™ SE and TactiCath™ SE are 4.0 and 3.5 mm, respectively).^5^ When we use a larger ablation tip catheter, a 30 W ablation might be underpowered because of the difference in the tip sizes.^5^ Therefore, the ablation power in protocol 2 was performed at 40 or 50 W. The RF energy was delivered with a combination of 2 power steps (40 and 50 W), 3 CF steps (10, 30, and 50 g), and 6 duration steps (10, 20, 30, 40, 50, and 60 s [same result of protocol 1]). Like protocol 1, ablation was continued even when a steam pop occurred. The experiment was repeated 10 times at each setting and all data were analyzed.

### 2.4 Statistical analysis

The statistical analyses were performed with GraphPad Prism9 software (GraphPad Software Inc.) and the Statistical Package for the Social Sciences for Windows (SPSS, version 27). All variables are expressed as the mean ± SD. Statistically significant differences were identified using a one‐way analysis of variance (ANOVA) with a Tukey‐Kramer post hoc analysis. The Cochran‐Armitage test was used for the trend analyses. A receiver‐operating characteristic (ROC) curve analysis and the area under the ROC curve were used to evaluate the cut‐off level to predict steam pops. A correlation analysis was performed using Pearson's correlation analysis (*r*). A nonlinear regression analysis or simple linear regression analysis was performed to investigate the relationship between the RF delivery time and lesion formation (lesion depth, lesion diameter, and lesion volume). A value of *p* < .05 was considered statistically significance, unless specified otherwise.

## 3 RESULTS

A total of 180 RF lesions were created in the protocol 1 study. The lesion formation, ablation parameters, and incidence of steam pops with the TactiFlex™ SE catheter and FlexAbility™ SE catheters were analyzed. A total of 300 RF lesions were created in the protocol 2 study. The time course changes were analyzed for those 300 RF lesions and for the 60 RF lesions in protocol 1.

### 3.1 Ablation characteristics of the TactiFlex™ SE catheter and FlexAbility™ SE catheter

**FIGURE 2:**
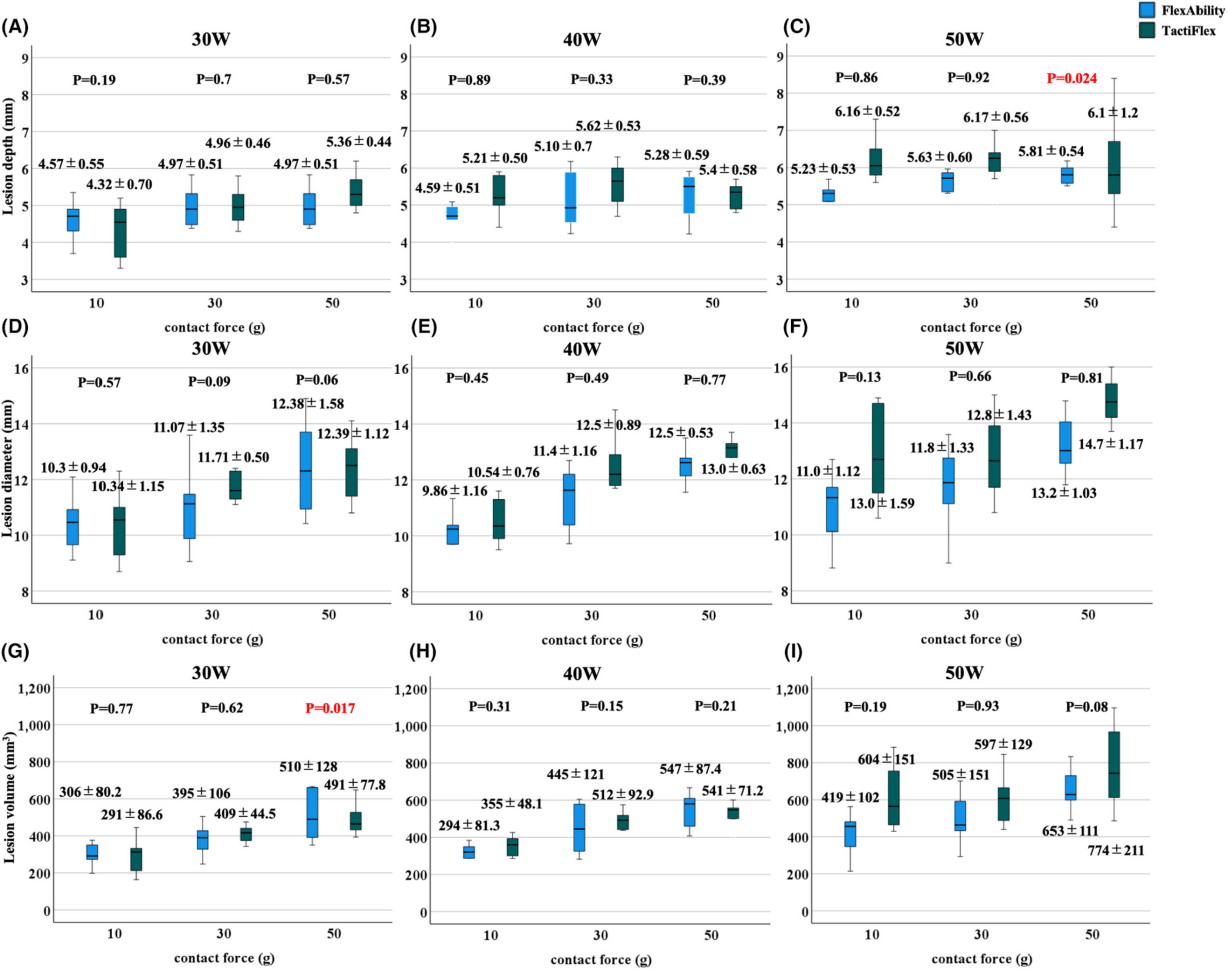
(A–F) Relationship between the CF and maximum lesion depth, maximum diameter, and lesion volume for each RF power and constant energy application duration (60 s) for each catheter. There were no significant differences between the two catheters regarding the lesion formation among each CF setting under each RF power setting except for the lesion depth with a 50 W/50 g setting and the lesion volume with a 30 W/50 g setting. CF, contact force; RF, radiofrequency.

A cross‐sectional comparison of the lesion size with TactiFlex™ SE and FlexAbility™ SE in protocol 1 revealed that, except for the lesion depth for 50 g and 50 W and lesion volume for 50 g and 30 W settings, there were no significant differences in the lesion depth, diameter, and volume between two catheters (Figure 2).

**FIGURE 3:**
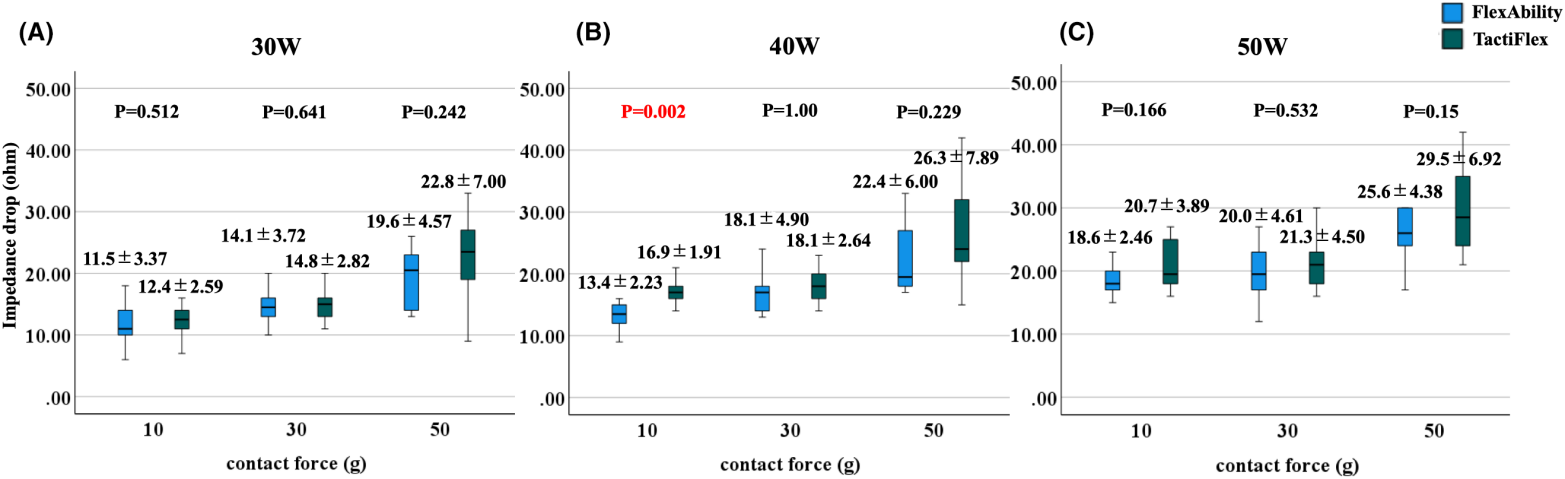
(A–C) Relationship between the impedance drop for each RF power and contact force setting for each catheter. There were no significant differences between the two catheters except for the 40 W/10 g setting. RF, radiofrequency.

**FIGURE 4:**
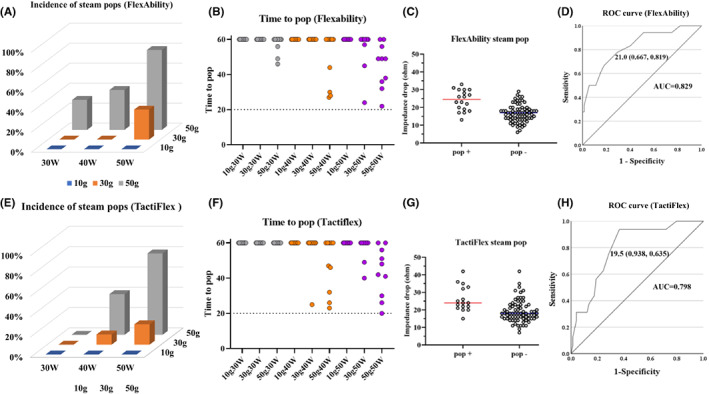
(A and E) Incidence of steam pops with each ablation setting using the FlexAbility™ SE and TactiFlex™ SE catheters. The incidence of steam pops was particularly higher with a CF of 50 g than with a CF of 10 or 30 g. There were no significant differences between the two catheters. (B and F) The time to the pop for each ablation setting using the FlexAbility™ SE and TactiFlex™ SE catheters. The time to the pop for the two catheters did not significantly differ. (C and G) The impedance drops with steam pops were significantly greater than those without steam pops for both catheters. (D and H) The ROC curve for the steam pops and impedance drop for the two catheters. The AUC was 0.798 and 0.829 for TactiFlexTM SE and FlexAbilityTM SE, respectively. The sensitivity/1‐specificity was greatest for an impedance drop of 19.5 and 21.0 Ω with a sensitivity of 0.667 and 0.938 and specificity of 0.819 and 0.635 for TactiFlexTM SE and FlexAbilityTM SE, respectively. CF, contact force; AUC, area under the curve; ROC, receiver operator characteristics.

No significant differences between the two catheters were observed regarding the impedance drops, except for that with the 10 g and 40 W setting (Figure 3A–C). There was no significant difference in the incidence of steam pops (Figure 4A,E; TactiFlex™ SE vs. FlexAbility™ SE, 16/90 [17.8%] vs. 18/90 [20.0%], *p* = .566). There was a higher incidence of steam pops with FlexAbility™ SE (FlexAbility™ SE; 50 g [15/30] vs. 10 g [0/30], 30 g, [3/30], *p* < .001, *p* < .001; TactiFlex™ SE; 50 g [12/30] vs. 10 g [0/30], 30 g [3/30], *p* < .001, *p* = .015). When we focused on lesions that popped during ablation, the time to the pop for the two catheters did not significantly differ (Figure 4B,F; TactiFlex™ SE catheter vs. FlexAbility™ SE; 50g40W, 34.8 ± 11.2 s vs. 32.3 ± 7.9 s, *p* = 0.713; 30 g 50 W, 44.5 ± 6.37 s vs. 42 ± 16.7 s, *p* = .859; 50 g 50 W, 39.3 ± 12.7 s vs. 41.4 ± 11.3 s, *p* = .729). For both products, the impedance drop for those with steam pops was significantly greater than for those without steam pops (Figure 4C,G, steam pop (+) vs. steam pop (−); TactiFlex™ SE, 26.3 ± 7.25 Ω vs. 19.0 ± 6.27 Ω, *p* < .001; FlexAbility™ SE 24.0 ± 5.80 Ω vs. 16.7 ± 4.87 Ω, p < 0.001). The ROC curves for the steam pops and impedance drops of the two products are shown in Figure 4D (TactiFlex™ SE) and 4H (FlexAbility™ SE). The AUC was 0.798 and 0.829 for TactiFlex™ SE and FlexAbility™ SE, respectively. The Youden index was greatest at an impedance drop of 19.5 Ω with a sensitivity of 0.938 and specificity of 0.635 for TactiFlex™ SE and 21.0 Ω with a sensitivity of 0.667 and specificity of 0.819 for FlexAbility™ SE, respectively.

### 3.2 Relationship between the RF delivery time and lesion formation for the TactiFlex™ SE catheter

**FIGURE 5:**
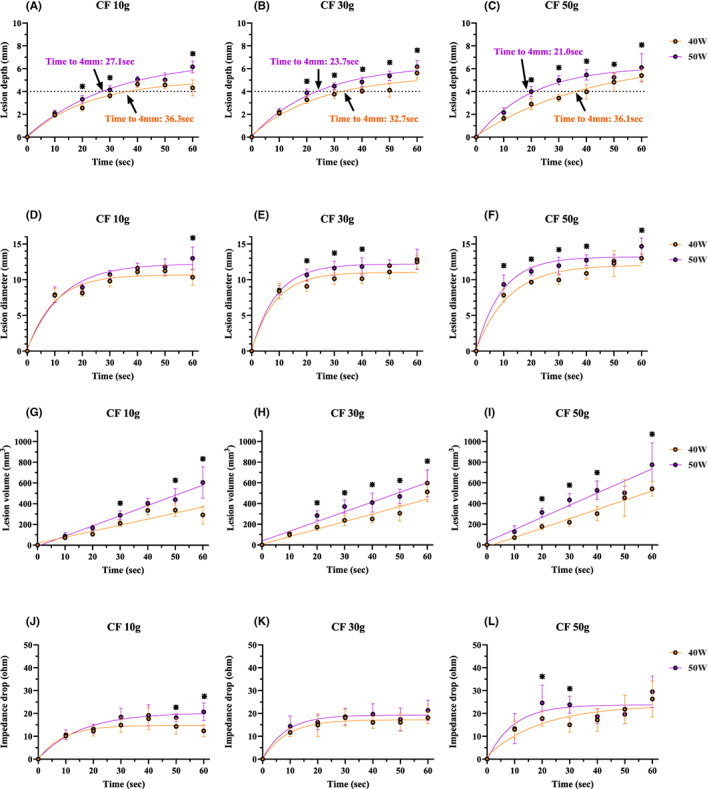
Relationship between the RF delivery time and lesion depth (A–C), lesion diameter (D–F), and lesion volume (G–I) under each ablation setting. A nonlinear, time‐dependent increase in the lesion depth and lesion diameter was observed for all power and CF settings. A linear, positive correlation between the RF delivery time and lesion volume was observed for all power settings and CF settings. The asterisks show the significant differences between 40 and 50 W for the ablation time. (J–L) Correlation between the impedance drop and RF delivery time. A statistically significant difference between the 40 and 50 W power settings is shown by the asterisk (*). A time‐dependent increase in the impedance drop was observed with all power and CF settings. CF, contact force; RF, radiofrequency.

A nonlinear, time‐dependent increase in the lesion depth (Figure 5A–C) and lesion diameter (Figure 5D–F) was observed for all power and CF settings (Lesion depth: 40 W 10 g; *R* ^2^ = 0.9281, 40 W 30 g; *R* ^2^ = 0.9020, 40 W 50 g; *R* ^2^ = 0.9182, 50 W 10 g; *R* ^2^ = 0.9531, 50 W 30 g; *R* ^2^ = 0.9561 and 50 W 50 g; *R* ^2^ = 0.9210, Figure 5A–C. Lesion diameter: 40 W 10 g; *R* ^2^ = 0.9285, 40 W 30 g; *R* ^2^ = 0.9174, 40 W 50 g; *R* ^2^ = 0.9230, 50 W 10 g; *R* ^2^ = 0.9310, 50 W 30 g; *R* ^2^ = 0.9495 and 50 W 50 g; *R* ^2^ = 0.9372, Figure 5D–F.).

In contrast, linear, positive correlations between the RF delivery time and lesion volume were observed for all power settings (40 W 10 g; *R* ^2^ = 0.7735, 40 W 30 g; *R* ^2^ = 0.8283, 40 W 50 g; *R* ^2^ = 0.8352, 50 W 10 g; *R* ^2^ = 0.8719, 50 W 30 g; *R* ^2^ = 0.8536 and 50 W 50 g; *R* ^2^ = 0.8257, Figure 5G–I).

**FIGURE 6:**
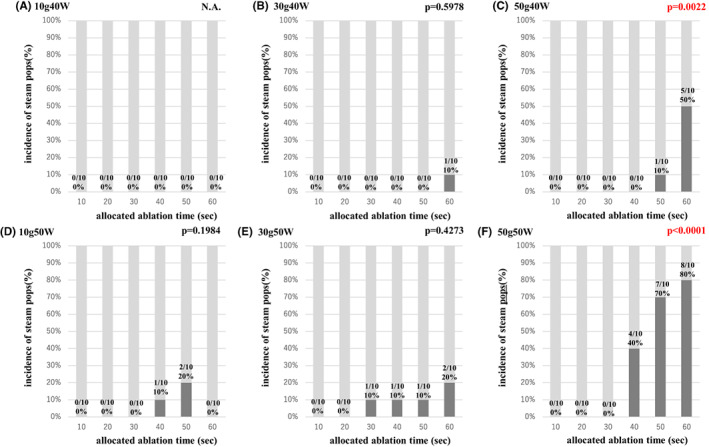
Incidence of steam pops under each ablation setting. (A–C) An ablation power with 40 W and (D–C) ablation power with 50 W. The longer ablation deliveries for the 50 g ablation had a higher incidence of steam pops.

Figure 6A–F shows the incidence of steam pops under each ablation setting. A trend analysis was performed to investigate the incidence of steam pops. The longer ablation delivery groups with the 50 g ablation had a higher incidence of steam pops.

## 4 DISCUSSION

### 4.1 Major findings

The major findings of our study were as follows:

(1) The lesion formation with the TactiFlex™ SE catheter and FlexAbility™ SE catheter was almost similar for the various CF and wattage settings under a 60 s ablation (Figure 2). There was no significant difference in the impedance drop between these two catheters under most ablation settings (Figure 3). (2) The incidence of steam pops did not exhibit a significant difference between the TactiFlex™ SE catheter and FlexAbility™ SE catheter. A higher CF was related to a higher incidence of steam pops (Figure 4A,E). The impedance cutoff level to predict steam pops was similar between the two catheters (Figure 4C,D,G,H). (3) Compared with the 40 W ablation, the 50 W ablation could create a greater lesion (Figure 5). A longer duration ablation with a higher CF setting had a higher incidence of steam pops (Figure 6). Monitoring impedance drops is important to prevent steam pops with a high‐power ablation.

### 4.2 Comparison of the lesion formation with the TactiFlex™ SE catheter

Compared with other previous reports, the maximum lesion depth, diameter, and lesion volume created with the TactiFlex™ SE catheter were smaller than that with the other 3.5 mm tip catheters.^5^, ^13^, ^16^ For instance, the maximum lesion depth, diameter, and lesion volume for the TactiFlex™ SE catheter with 30 W and a 10 g CF were 4.32 ± 0.70 mm, 10.34 ± 1.15 mm, and 291 ± 86.6 mm^3^, respectively, which were smaller than those with a 3.5 mm tip catheter (TactiCath™ SE; depth 7.3 mm, diameter 10.2 mm, and volume 431 mm^3^),^5^ suggesting a possibility that the relatively long tip length of TactiFlex™ SE (4.0 vs. 3.5 mm) contributed to a smaller current density, resulting in low resistive heating, and thus a smaller lesion size than that of the TactiCath™ SE catheter. If this were true, it would indicate that a higher power is needed with TactiFlex™ SE to create a lesion comparable to one with TactiCath™ SE.

Our study compared the lesion formation with the TactiFlex™ SE catheter and its predecessor the FlexAbility™ SE catheter and showed that these were almost similar with the various CF and wattage settings under a 60 s ablation (Figure 2). We speculated that, regardless of the presence of a CF sensor, the current density of the same mesh‐shaped catheter would be similar and would finally create a similar ablation lesion.^12^ Likewise, it is reasonable to expect that the same ablation tip would create similar impedance changes. Moreover, a previous report noted that a parallel catheter placement with FlexAbility™ SE produced larger lesions compared with a perpendicular catheter placement.^5^ That previous fact suggested the possibility that a lesion creation of the TactiFlex™ SE catheter with a parallel catheter placement would be larger than that with a perpendicular catheter placement.

### 4.3 Relationship between the steam pops and impedance drops

Compared with the 10–30 g ablation, the 50 g ablation had a higher incidence of steam pops (Figure 4A,E). The mesh‐shaped tips of the two catheters we tested both had a slit structure. A higher CF with a perpendicular setting should deform the tip, narrowing the gaps between the slits and thus shortening the tip length, which may increase the current density in response to a decrease in the tip surface area. Those characteristics of the mesh‐shaped catheters would be related to a higher incidence of steam pops at higher CF settings. If this interpretation were to be true, a prolonged ablation at a high CF would be more likely to result in a steam pop (Figure 6C,F). We suggest that the CF should be controlled in order not to exceed 30 g with TactiFlex™ SE.

Although a previous report noted that impedance drops are not necessarily related to the steam pops,^16^ our study revealed that the impedance drop was useful for predicting steam pops, and the cut‐off values for the steam pops were 19 Ω (TactiFlex™ SE) and 21.0 Ω (FlexAbility™ SE), respectively. This is in good agreement with another report that suggested that an impedance drop of >18 Ω predicts a steam pop with the NaviStar ThermoCool (Biosense Webster).^17^, ^18^ Care should be given to monitoring for impedance drops.

### 4.4 Optimal RF delivery wattage and time for the pulmonary vein isolation

Pulmonary vein isolation (PVI) is a cornerstone strategy for the treatment of AF.^19^ Late reconnections caused by an insufficient lesion lead to the recurrence of AF.^19^, ^20^ regarding this point, the creation of transmural lesions is indispensable for AF ablation. However, a longer ablation is related to ablation complications such as steam pops or collateral damage to the esophagus.^1^, ^21^, ^22^ Previous studies noted that the average wall thickness of the atrial tissue was 2.0 mm and the maximum thickness was less than 4.0 mm.^23^, ^24^ Considering these histological characteristics, an ablation lesion of 4.0 mm is considered to be needed for transmural lesions for the PVI. We focused on the RF delivery time to achieve a lesion depth of 4 mm. Our study revealed that the ablation time to create 4 mm lesions was 32.7–36.3 s with a 40 W ablation and 21.0–27.1 s with a 50 W ablation. In clinical RF ablation, the CF is generally controlled between 10 and 30 g, hence the optimal ablation time for the TactiFlex™ SE catheter for a PVI is about 30 s with a 40 W and 20 s with 50 W setting. However, a higher power ablation is related to a higher incidence of steam pops, which is related to cardiac tamponade.^25^ Compared with the TactiCath™ SE catheters, the incidence of steam pops with mesh‐shaped tip catheters was smaller because of the larger tip.^26^ But care should be given to monitoring the general impedance during RF applications.

### 4.5 Limitations

There were several limitations to our study. Firstly, this study was performed with an ex vivo experimental model. Various conditions differed between the RF ablation in this experimental model and the real clinical RF ablation. The reaction of living tissue and the CF changes because of the heartbeats, respirations, or human anatomy, were not considered in our results. Especially, the incidence of steam pops was generally lower in the in vivo model than ex vivo model. Secondly, the catheter orientation in our study was performed with a perpendicular setting. Our results might have differed from that with other catheter angles.

## 5 CONCLUSION

The lesion formation and incidence of steam pops with TactiFlex™ SE and FlexAbility™ SE were largely similar for the same experimental settings. When we used TactiFlex™ SE to create a lesion of which the size was comparable to that with the TactiCath™ SE, a higher power or longer duration was required. In this context, to avoid steam pops, a careful CF control in order not to exceed 30 g in addition to monitoring for impedance drops may be needed.

## AUTHOR CONTRIBUTIONS

Hitoshi Mori and Ritsushi Kato contributed to the study conception and design. Kazuhisa Matsumoto, Wataru Sasaki, Daisuke Kawano, Naomichi Tanaka, and Masataka Narita contributed to the data collection and data analysis. Kenta Tsutsui, Yoshifumi Ikeda, Takahide Arai, Shintaro Nakano, and Kazuo Matsumoto contributed to the manuscript revision and study supervision.

## CONFLICT OF INTEREST STATEMENT

All authors declare no conflict of interest associated with this study.
